# The Impact of Task Demands on Fixation-Related Brain Potentials during Guided Search

**DOI:** 10.1371/journal.pone.0157260

**Published:** 2016-06-10

**Authors:** Anthony J. Ries, Jon Touryan, Barry Ahrens, Patrick Connolly

**Affiliations:** 1 Human Research and Engineering Directorate, U.S. Army Research Laboratory, Aberdeen Proving Ground, Maryland, United States of America; 2 College of Engineering and Computing, Nova Southeastern University, Fort Lauderdale, Florida, United States of America; 3 Teledyne Scientific Company, Durham, North Carolina, United States of America; University of Waterloo, CANADA

## Abstract

Recording synchronous data from EEG and eye-tracking provides a unique methodological approach for measuring the sensory and cognitive processes of overt visual search. Using this approach we obtained fixation related potentials (FRPs) during a guided visual search task specifically focusing on the lambda and P3 components. An outstanding question is whether the lambda and P3 FRP components are influenced by concurrent task demands. We addressed this question by obtaining simultaneous eye-movement and electroencephalographic (EEG) measures during a guided visual search task while parametrically modulating working memory load using an auditory N-back task. Participants performed the guided search task alone, while ignoring binaurally presented digits, or while using the auditory information in a 0, 1, or 2-back task. The results showed increased reaction time and decreased accuracy in both the visual search and N-back tasks as a function of auditory load. Moreover, high auditory task demands increased the P3 but not the lambda latency while the amplitude of both lambda and P3 was reduced during high auditory task demands. The results show that both early and late stages of visual processing indexed by FRPs are significantly affected by concurrent task demands imposed by auditory working memory.

## Introduction

Visual search is an essential component of everyday human behavior and requisite for a number of real-world tasks. There is a large body of work, conducted over many decades, dedicated to exploring and understanding the mechanisms of visual search [1]. A significant portion of this work has focused on evaluating EEG responses during covert visual search whereby participants must search for a target without making eye movements [2–4]. An advantage of covert visual search in EEG studies is that it limits the non-neural sources of noise produced by eye movements which have a much greater magnitude with respect to the neural signals of interest. A shortcoming of this approach, however, is that it restricts our understanding of the neural correlates of visual perception during overt visual search, that is, visual search tasks performed with eye movements. Recent methodological developments have begun to address this shortcoming by isolating ocular and brain-based activity during tasks that are typically performed with eye movements (e.g. free-viewing visual search).

Combining simultaneous recordings of EEG and eye-tracking data offers researchers the opportunity to implement tasks that embrace rather than limit eye movements. Within this framework, eye-tracking data are used to determine the onset and offset of saccades (and corresponding fixations) as well as their time-course and magnitude, thereby providing unique events for time-locking in the EEG data. This approach provides a powerful method to evaluate neural processing of visual information by extracting and averaging EEG during periods of eye-fixation to isolate fixation-related potentials (FRPs). Additionally, modern eye trackers provide highly accurate coordinates of the gaze position relative to the display, enabling the analysis of neural evoked activity as a function of fixated visual features and locations. Various studies ranging from reading to free-viewing visual search have demonstrated the utility of using FRPs to evaluate the neural correlates of vision at multiple levels of processing (e.g. [5,6].

The earliest and most prominent brain potential following fixation onset is commonly referred to as the lambda response and reflects the afferent flow of visual information at fixation to visual cortex [7–9]. The fixation-locked lambda response is a large positive potential similar to the visual stimulus-locked P1 event-related potential (ERP) [8,10,11]. As with the visual P1 ERP, the lambda response is affected by low level visual features such as luminance, contrast and spatial frequency [12–16]. Specifically, decreases in target luminance show decreased amplitude and increased latency in the lambda response (Gaarder et al., 1964, Scott et al., 1967). Both the lambda response and the visually-evoked P1 ERP component show similar scalp topographies with source dipoles located in striate/extrastriate visual cortex [8,11,17,18]. Differences between these two components are primarily seen when directly comparing their latency and amplitude. When evoked by the same stimulus, the lambda response generally peaks earlier and has a larger amplitude compared to the visually evoked P1 ERP [6,11].

More recent investigations have revealed longer latency FRP components that distinguish target from non-target fixations in visual search. Using a fixed array of Landold C’s in a guided sequential search task, Brouwer et al 2013 showed that FRPs time-locked to target fixations produced a large late cognitive potential similar to the P3 ERP. This finding was extended by Kaunitz et al, 2014 who used a free viewing search paradigm in natural scenes. They found larger P3 FRP amplitude to target faces with respect to non-target faces. These and other studies have shown that P3 FRP is very similar to the P3 ERP in terms of onset, duration and offset when comparing the same target stimulus in fixation locked with respect to stimulus locked paradigms. However, there appears to be a slight difference in the spatial distribution of the P3 FRP compared with its stimulus locked counterpart [6,19]. Given the late stages of processing indexed by the P3, tasks are generally designed to artificially lengthen fixations to help reduce signal overlap from preceding or following saccades/fixations.

While several studies have investigated the effects of low-level visual features and target/non-target characteristics on FRP components, it is still unclear if FRPs are affected by task demands. Visual search behavior is regularly coupled with concurrent tasks requiring access to the limited processing capacity of working memory. For example many people converse on mobile phones while driving, which is known to significantly impair driving performance [20]. This requires processing of both the visual aspects of driving such as searching for potential obstacles as well as the active maintenance of auditory information from the conversation. Investigating fixation-related neural activity under different levels of task demands will help reveal how top-down influences affect the amplitude and time-course of the well-established FRP components at both the early (lambda response) and later (P3) processing stages.

Findings from the ERP and fMRI literature provide insight into how task-related, top-down influences, specifically the processing demands imposed by working memory load, may affect FRPs. Generally it has been shown that visual P1 and P3 amplitudes decrease while P3 latency increases under conditions of high processing demands [21–24]. More specifically it has been shown that manipulating working memory load in one task can affect multiple levels of neural processing in another. For example, in a study by [24] subjects performed an arrow flanker task either alone or while performing a Sternberg task with high or low working memory load. The results showed decreased P1 ERP amplitude to flanker stimuli in the dual with respect to single task as well as decreased P3 ERP amplitude with increased working memory load for incongruent flanker stimuli. Others have shown that variations of working memory load in one modality can affect early sensory evoked responses in another. This was evidenced in a study showing significantly decreased auditory brainstem responses as a function of increased visual working memory load [25]. Similarly, using an auditory working memory task [26] showed that visual blood oxygen level dependent (BOLD) responses in occipital cortex, as measured with fMRI, decreased as auditory working memory load increased. Together the results provide evidence for a resource-sensitive mechanism that modulates processing both within and across modalities; however it is still unclear to what degree task demands imposed by working memory load affect components of the FRP.

Here we developed an experimental paradigm that allowed us to investigate both early and late components of the FRP, namely the lambda response and P3, as a function of working memory load while controlling for saccade magnitude and fixation duration. Subjects engaged in a guided visual search task while simultaneously performing an auditory N-back task. Using an auditory working memory task allowed us to assess the impact of cognitive demands on FRPs while ensuring that any effects of task load on visual processing were not due to within-modality sensory conflicts. We hypothesized that the amplitude of the lambda potential would decrease with increasing working memory load. Additionally, we predicted that increases in auditory working memory load would result in decreased P3 amplitude and increased P3 latency.

## Materials and Methods

### Participants

Fourteen participants volunteered for the study; all participants we right-handed males with an average age of 32.8 years. All participants had 20/20 vision or corrected to 20/20 vision. The voluntary, fully informed consent of the persons used in this research was obtained in written form. The document used to obtain informed consent was approved by the U.S. Army Research Laboratory’s Institutional Review Board (IRB) in accordance with 32 CFR 219 and AR 70–25, and also in compliance with the Declaration of Helsinki. The study was reviewed and approved (approval # ARL 14–042) by the U.S. Army Research Laboratory’s IRB before the study began.

### Stimuli and Procedure

**Fig 1:**
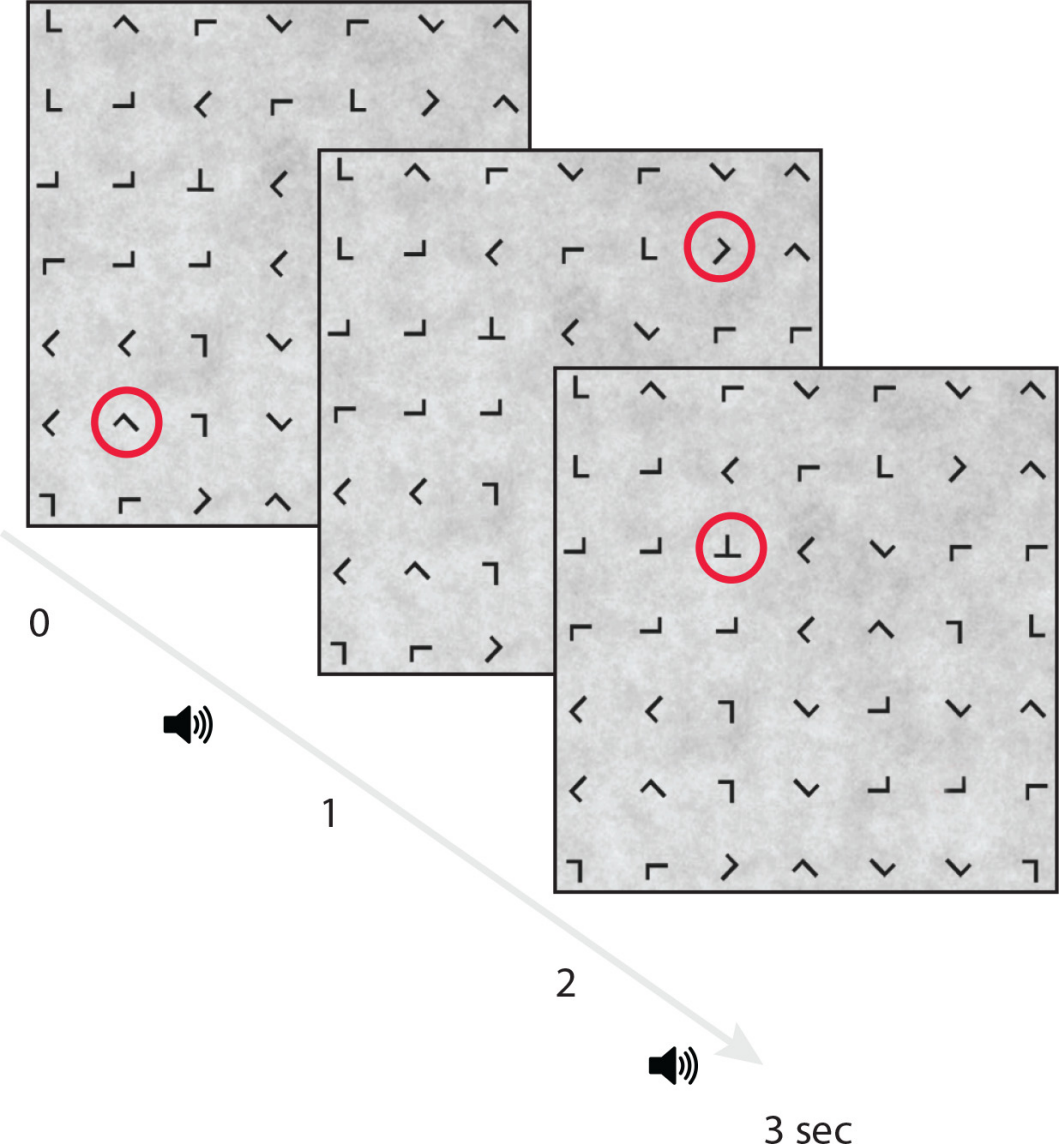
Guided visual search task. Red annuli changed location on the display search grid indicating the character subjects should fixate. The guided fixation task was performed alone, while ignoring binaurally presented numbers or using the numbers in an N-back task (0, 1, or 2-back). Fixations were guided to a new location every second and auditory information was presented every two seconds with a 500ms offset from the onset of the visual task. The first two fixations in the current example are non-target “L” characters and the last fixation depicts a target “T” character.

Participants performed an overt, guided visual search task on a 7x7 grid (23.9° x 23.9° visual angle) of equiluminant, equally spaced, and variably oriented ‘T’ or ‘L’ characters (1.1° visual angle) presented on a low contrast 1/f noise background (Fig 1). The task was performed on Dell 1920x1280 LCD monitor from a viewing distance of 65cm. Eye fixations were guided across the grid by a red annulus (2.3° visual angle, RGB 255, 25, 0) that randomly surrounded one of the characters. The red annulus remained visible for one-second after which it moved to a different character location. The characters on the grid changed every 5 seconds. Participants were instructed to saccade to and fixate on the character in the center of the red annulus and make a button press with their left hand using an Xbox 360 controller only when a “T” (visual target) was present. Visual target characters appeared on 10% of trials. Participants were instructed to maintain fixation on the character until the next red annulus appeared. All red annuli surrounding a non-target “L” were at least two characters from a “T” if it was present on the grid which prevented participants from detecting targets in their peripheral vision. The guided visual search task was performed alone, while ignoring binaurally presented digits (numbers 0–9), or while using the auditory digits in a 0, 1, or 2-back working memory task. The digit ‘0’ was only used in the 0-Back condition where it served as the auditory target. Auditory stimuli had an average duration of 290ms and were presented every 2 seconds with a 500ms offset from the red annulus in the visual search task to prevent simultaneous auditory/visual events. The auditory stimuli were presented through two equally-spaced speakers to the right and left of the computer monitor. Participants made a button press with their right hand for auditory targets, which occurred on 20% of auditory trials. Thus, the same number of targets appeared in both the auditory and visual task conditions on each block. Participants performed two consecutive blocks of the same condition (Silent, Ignore, 0-back, 1-back, 2-back) with condition order counterbalanced. Each block lasted three minutes and twenty seconds. Self-paced rest periods were given between each block. Participants were given practice in each N-back condition prior to experimental data collection until they reached above chance performance.

### EEG and Eye Tracking

EEG data were sampled at 512Hz from 64 active scalp electrodes using the Biosemi Active Two system (Amsterdam, Netherlands). External electrodes were placed at the outer canthus of each eye, above and below the orbital fossa of the right eye and on the right and left mastoid. All electrode impedances were below 25kΩ and referenced to the Common Mode Sense (CMS) active electrode (http://www.biosemi.com/faq/cms&drl.htm). Eye tracking data were sampled at 250Hz using the SMI RED 250 system (Teltow, Germany). A 15-point calibration was performed prior to the practice and experimental blocks. A post hoc model was fit to the eye tracking data for each participant to increase accuracy of the gaze position estimate. Briefly, we used the expected eye position (i.e., location of the red annulus) to fit a quadratic regression model for both the X and Y gaze position vectors. This resulted in a substantial improvement in the estimate of gaze position, especially at the periphery of the search grid.

### Data Processing

Data from the visual presentation, behavioral responses, EEG, and eye tracking acquisition systems were synchronized using custom software by aligning independently injected events into each data stream. EEG and subsequent FRP data were processed using EEGLAB and the ERPLAB plugin [27,28]. EEG were first re-referenced to the average of the right and left mastoids and filtered using a second order Butterworth filter with a 1–40Hz passband. The use of a mastoid referencing has been used previously to extract and analyze fixation related potentials [6,19,29–32]. Other reference approaches such as the reference electrode standardization technique (REST) may be appropriate when trying to determine the underlying neural sources of FRPs [33–35] Infomax independent component analysis (ICA) was then performed to identify sources of ocular artifact. Blinks were identified using custom software and saccades and fixations were detected in the eye-tracking data using the velocity-based algorithm provided in the EYE-EEG extension (*http*:*//www2*.*hu-berlin*.*de/eyetracking-eeg*) for EEGLAB [5,36]. Blinks, saccades and fixations were added as unique events in the EEG data structure. Saccades and fixations were detected using a velocity factor of 6, minimum saccade duration of 20ms, minimum fixation duration of 350ms and only the largest saccade and corresponding fixation was kept if two saccades occurred within 350ms. While the eye remains relatively stable during a fixation there still remains eye movement related muscle activity that overlaps with neural response. In order to remove residual noise from ocular muscle artifact we rejected independent components based on their covariance with the eye-tracking data [37]. Components with a ratio of 1.1 or greater were removed. After ocular artifact removal, the continuous EEG were epoched, time-locked to fixation onset with 500ms before and 1000ms after each fixation. Next we rejected fixation-locked epochs if peak to peak activity surpassed a 100μV threshold using a 100ms window size and a window step of 50ms. FRPs were created for each condition (Silent, Ignore, 0-Back, 1-Back, 2-Back) by averaging the fixation-locked EEG epochs. Only FRPs having fixations within 3° of the red annulus, behaviorally correct, not immediately preceded or followed by an auditory target trial, and not preceded or followed by a blink, boundary event or saccade/fixation within 500ms were included in the final analysis and are hence referred to as valid trials. Greenhouse-Geisser statistics are reported along with Sidak correction for multiple comparisons when appropriate [38,39].

## Results

### Behavior

**Fig 2:**
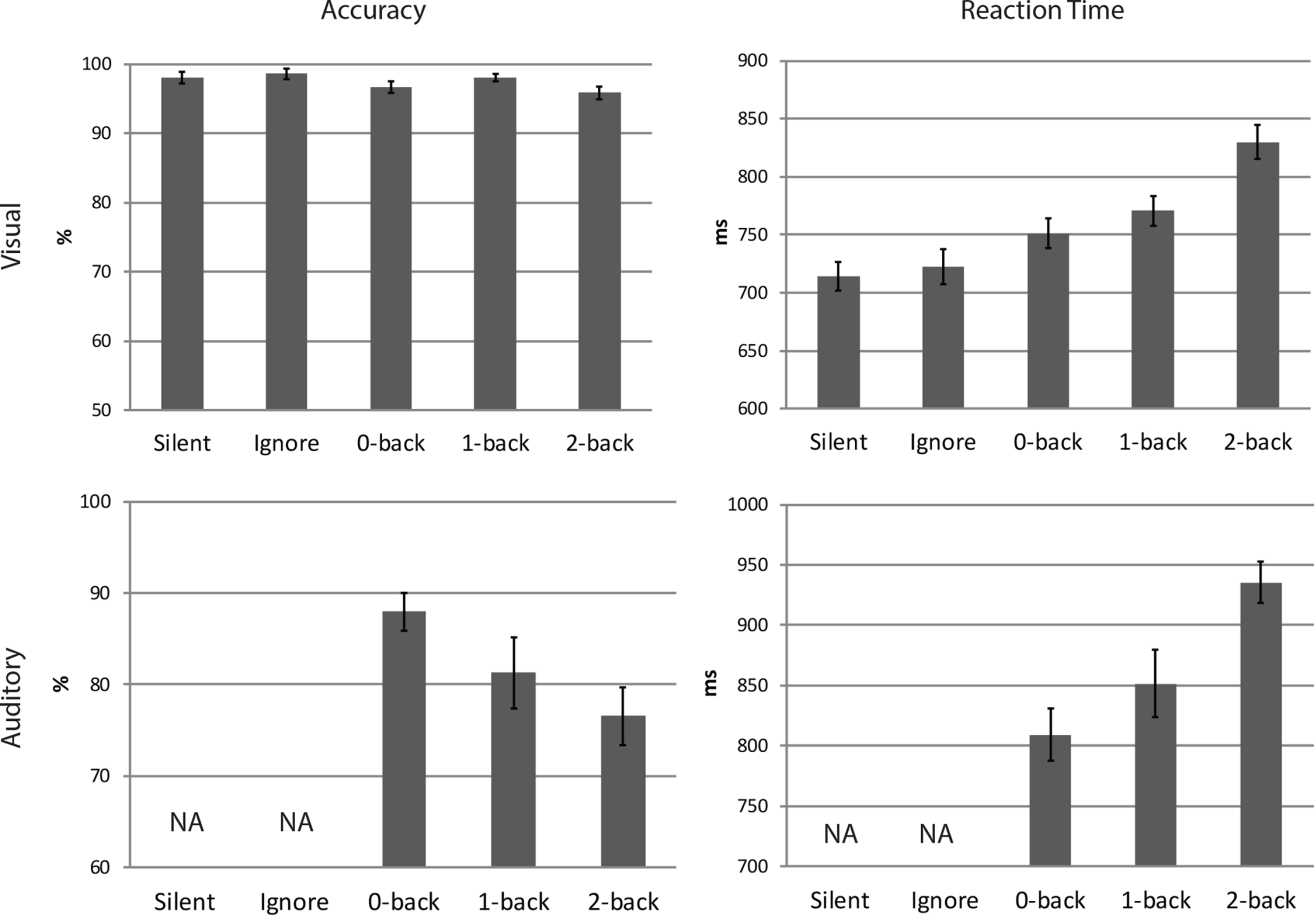
Behavioral performance in the visual search and auditory N-back tasks. Error bars equal +/- SE. NA = not applicable.

Reaction time and accuracy were analyzed separately for the visual and auditory tasks using a one-way repeated measures ANOVA. The primary factor was auditory task load. This factor had five levels in the visual task (Silent, Ignore, 0-Back, 1-Back, 2-Back), and three levels in the auditory task (0-Back, 1-Back, 2-Back). There was a trend for decreased accuracy in the visual search task as a function of auditory load; however this was not statistically significant *F*(2.68, 34.85) = 2.43, *p* = .088; *η*^2^ = .158. A highly significant effect of auditory load was found on reaction time in the visual search task *F*(2.73,35.47) = 29.29, *p* < .001; *η*^2^ = .69, showing that visual target reaction time increased as auditory task demands increased. After adjusting for multiple comparisons, significant differences were obtained between 2-Back and all other conditions in addition to Silent and 0-Back, Silent and 1-Back, and Ignore and 1-Back. Analysis of the auditory task showed significantly decreased accuracy and significantly increased reaction time as a function of auditory working memory load, *F*(1.61,20.97) = 6.73, *p* = .008; *η*^2^ = .34, *F*(1.6,20.8) = 17.62, *p* < .001; *η*^2^ = .58 respectively. Follow up comparisons showed auditory target accuracy was significantly different between 0-Back and 2-Back only. Significant differences between auditory target reaction time were found for 0-Back and 2-Back, and 1-Back and 2-Back. The behavioral results suggest that auditory working memory load had a negative impact on visual task performance especially at the highest load (2-Back). Likewise, these results show that participants were not favoring one task over the other as behavioral performance declined in both the visual and auditory tasks as auditory task demands increased (Fig 2). Behavioral data from each subject can be found in the Supplementary Information in S11 File.

### Eye Metrics

#### Saccade Magnitude

FRP studies using reading and visual search paradigms have shown that saccade magnitude affects the amplitude of the lambda response. Specifically, FRP amplitude increases with saccade magnitude [5,6,9,19]. Therefore, we first analyzed valid trials (see section 2 Materials and Methods) from each auditory load condition to determine if changes in FRP amplitude could be attributed to saccade magnitude. We ran a one-way ANOVA with auditory task load (Silent, Ignore, 0-Back, 1-Back, 2-Back) as the primary factor and found no significant differences in saccade magnitude F(2.84,36.86) = 0.76, p = .515, *η*^2^ = .06. Saccade magnitude was highly similar between auditory load conditions. Horizontal saccades occurred more often than vertical saccades and this pattern was not favorably different between auditory conditions (S1 Fig). The majority of saccades had amplitudes between 5° and 25° with an average saccade amplitude of 13.57°. Summary eye tracking data from each subject can be found in the Supporting Information in S11 File.

#### Fixation Reaction Time

**Fig 3:**
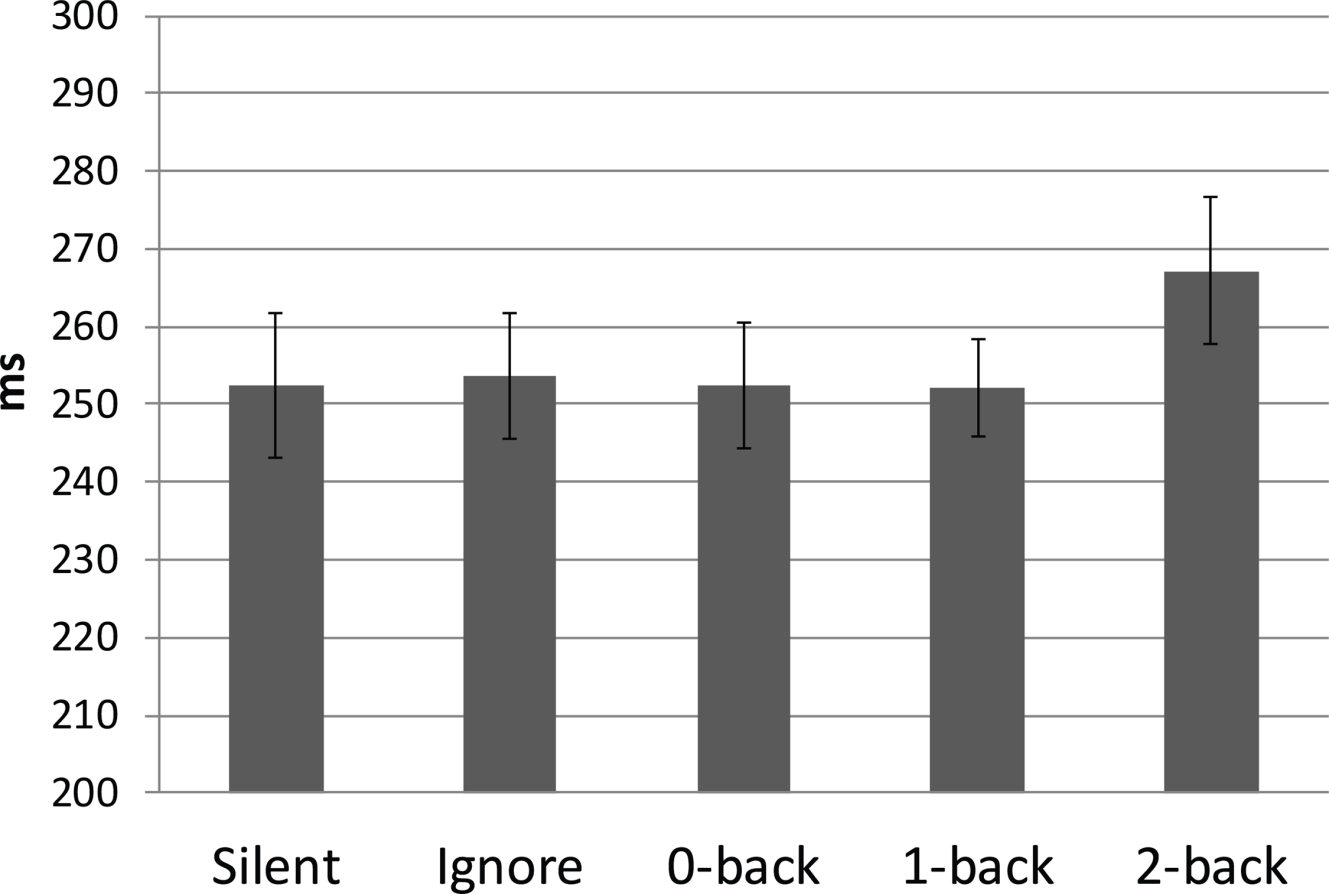
Fixation Reaction Time. Average latency between the onset of the red annulus and its subsequent fixation as a function of auditory load. Error bars equal +/- SE.

Fixation reaction time was calculated as the time between the onset of the red annulus and the onset of the subsequent fixation and analyzed using a one-way ANOVA with auditory task load (Silent, Ignore, 0-Back, 1-Back, 2-Back) as the primary factor. The main effect for task load was significant F(2.67,34.68) = 5.46, p = .005, *η*^2^ = .3 (Fig 3). Silent, Ignore, 0-Back, and 1-Back were all significantly faster when compared to the 2-Back condition after multiple comparison correction (all p < .05). No other comparisons were significant.

#### Fixation Duration

Fixation duration was analyzed using a two-way ANOVA with stimulus (target, non-target) and auditory task load as the primary factors. There was a trend for increased fixation duration on target compared to non-target stimuli, however, it was not statistically significant, F(1,13) = 3.28, p = .093, *η*^2^ = .202 (Table 1). The main effect of auditory task load and the stimulus by load interaction was not significant, F(1.32,17.19) = 0.64, p = .477, *η*^2^ = .047, and F(1.82,23.6) = 1.29, p = .291, *η*^2^ = .09 respectively. Thus, within the relevant eye metrics we found no systematic difference among task or stimulus conditions that could bias the FRPs.

**Table 1:** Target and non-target fixation duration by auditory load.

	Silent	Ignore	0-Back	1-Back	2-Back
**Target**	1.00 (.02)	0.97 (.01)	1.04 (.06)	0.97 (.02)	0.98 (.01)
**Non-target**	0.97 (.01)	0.98 (.01)	0.97 (.01)	.97 (.01)	0.95 (.01)

Note: standard error in parentheses

### FRPs

#### Lambda Potential

**Fig 4:**
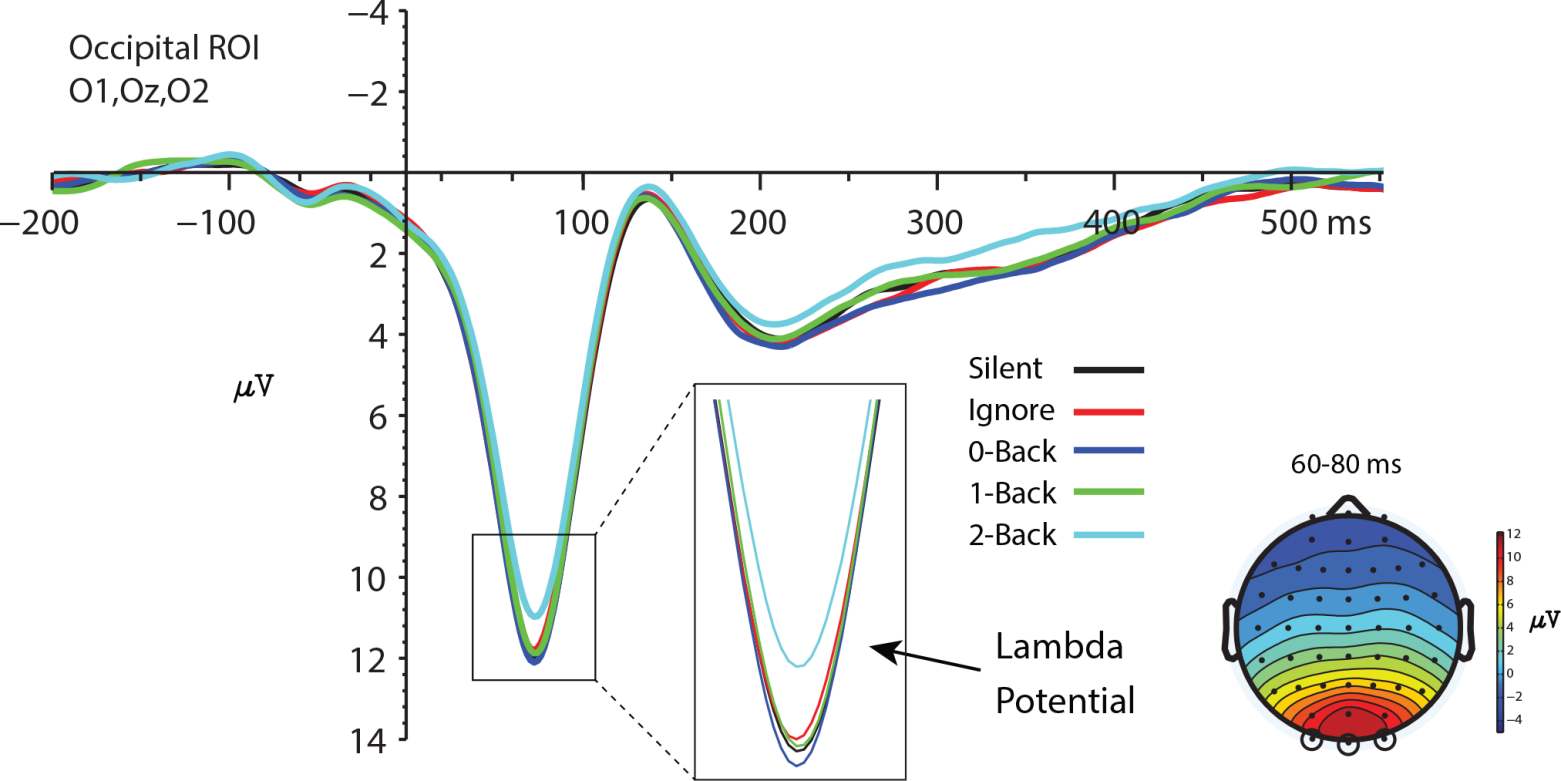
Lambda potential. Left—The non-target lambda potential obtained from the occipital ROI in each auditory condition. Right—Topographical voltage map highlighting the spatial distribution of the lambda response 60–80ms post fixation averaged across all non-target auditory conditions. Open circles indicate electrodes used in the ROI.

We focused only on valid non-target trials in the visual task that met the criteria for inclusion (see Materials and Methods). Grand average files can be found in the Supporting Information in S1–S5 Files. The non-target trials were chosen due to their relative frequency (compared with target trials) and hence better signal to noise. In a similar vein, we averaged data from electrodes O1, Oz, and O2 to create an occipital region of interest (ROI) for analysis. The average trial count with standard deviation in the Silent, Ignore, 0-Back, 1-Back, and 2-Back conditions was 159.6(40.1), 162.6(60), 150.6(58.7), 174.1(56.8), and 160.4(58.1) respectively. We evaluated both the peak latency and amplitude of the lambda potential (Table 2) using a one way repeated measures ANOVA with auditory task load as the main factor. The amplitude and latency of the lambda response was measured using the peak latency or average amplitude 60–80ms post fixation using -200 to -100ms pre-fixation baseline. No significant effect of auditory task load was found for the latency of the lambda response *F*(3.5,45.45) = 0.04, *p* = .997; *η*^2^ = .003 as the average peak latency was similar across conditions (72.5ms). However, there was a significant main effect of auditory task load on the amplitude of the lambda response *F*(2.68,34.84) = 5.37, *p* = .005; *η*^2^ = .292 (Fig 4).

**Table 2:** Average latency in milliseconds and amplitude in microvolts for the fixation-locked lambda response and P3.

	Silent	Ignore	0-Back	1-Back	2-Back
**λ Latency**	72.68 (1.6)	72.55 (1.7)	72.68 (1.6)	72.41 (1.4)	72.68 (1.5)
**λ Amplitude**	11.46 (0.8)	11.35 (0.8)	11.61 (0.7)	11.37 (0.8)	10.51 (0.8)
**P3 Latency**	505.86 (1.04)	517.79 (0.9)	526.86 (0.95)	546.86 (1.1)	588.71 (1.38)
**P3 Amplitude**	5.24 (0.06)	5.89 (0.05)	4.69 (0.04)	4.74 (0.06)	3.89 (0.04)

Note: standard error in parentheses

Follow up comparisons revealed significant amplitude differences between the Silent and 2-Back, Ignore and 2-Back, 0-Back and 2-Back. All other differences were not significant after multiple comparison corrections. Thus, while auditory load did not significantly affect the latency of the lambda response it did have a significant impact on the amplitude at the highest working memory load.

#### P3 Component

**Fig 5:**
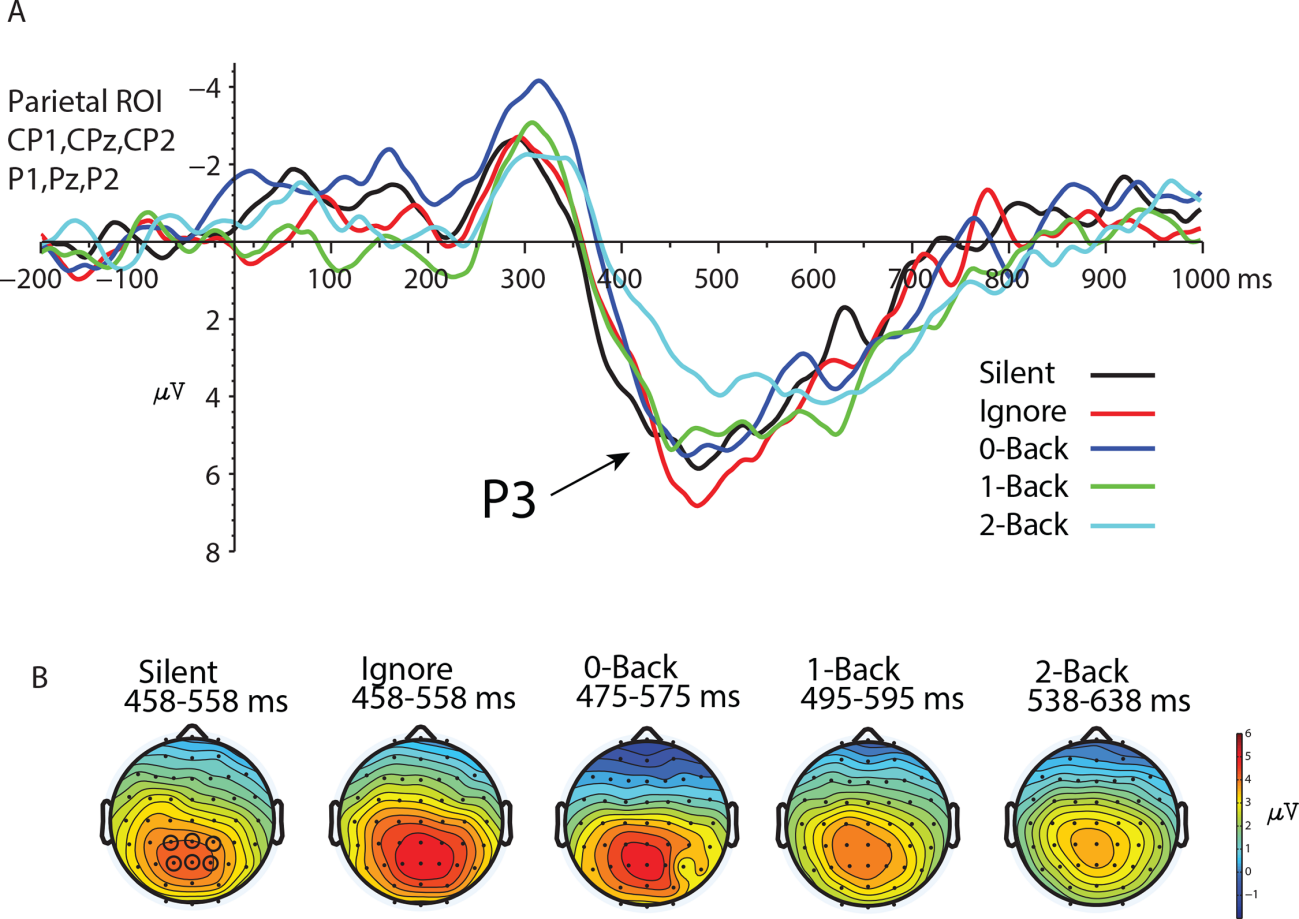
**P3 component of the FRP** A. Target–non-target difference waves time-locked to fixation onset. Data reflect the jackknifed results obtained from the parietal ROI for each auditory condition. B. Topographical voltage maps highlighting the distribution of the P3 response +/- 100ms around the 50% fractional area latency. Open circles indicate electrodes used in the ROI.

The latency of the P3 FRP component was analyzed using the fractional area latency measure combined with the jackknife approach due to the few number of trials and decreased signal to noise ratio afforded in target trials [40–42]. Grand average files can be found in the Supporting Information in S6–S10 Files. The analysis was performed on a parietal region of interest created from electrodes CP1, CPz, CP2, P1, Pz, & P2 (Table 2). We evaluated the 50% fractional area latency of the P3 FRP component using the target/non-target difference waves [43]. Latency values were entered into an ANOVA using the same factors as those used in the lambda potential. Here we found a significant main effect of load F (2.85,37.01) = 6.8, p = .003, *η*^2^ = .34 (Fig 5). The low frequency nature of the P3 component was likely attenuated due to the 1 Hz high-pass filter. Therefore, we reanalyzed the data using a 0.1Hz filter. The same result was obtained after analyzing the data processed with a 0.1Hz high-pass filter F(3.06,39.8) = 4.51, p = .008). The 50% fractional area latency significantly increased with auditory task demands. After correcting for multiple comparisons we found significant latency differences between the Silent and 2-Back, Ignore and 2-Back, and the 0-Back and 2-Back conditions. No other comparisons were significantly different after correction. No main effect of load was found for either P3 amplitude when evaluated +/- 100ms surrounding the 50% fractional area latency (F(2.97, 38.31) = 2.01, p = .12). Thus, while auditory load did not significantly affect the amplitude of the P3 response it did have a significant impact on the latency at the highest working memory load.

## Discussion

The current study evaluated the effect of auditory task demands on two components of the fixation related potential, the lambda response and P3. We employed a guided visual search task that was kept constant across varying levels of auditory task demands through an auditory N-back paradigm. Eye fixation events were detected from high-resolution (temporal and spatial) gaze position data and marked in the EEG record, allowing us to create fixation-related potentials as a function of auditory task load. As expected, the behavioral performance suffered in both the auditory and visual tasks as auditory working memory increased. However, our FRP analysis provides new evidence that both early and late stages of visual processing indexed by FRPs are significantly affected by changes in auditory working memory demand. Combining EEG and eye-tracking allowed us to measure the neurophysiological correlates of vision at multiple levels of processing without constraining eye-movements, thereby increasing the ecological validity of our findings.

### Lambda Response and the P3

The current study is not the first to evaluate the impact of auditory task demands on FRP responses. [44]), had participants perform a simulated driving task while performing either a verbal or spatial working memory task. The results showed a decrease in the amplitude of the lambda potential with increased verbal but not spatial working memory. However, this study used EOG to measure saccade amplitude which has an accuracy of approximately 2 degrees [45]. It is well known that changes in saccade magnitude correspond to changes in the amplitude of the lambda potential [5,6,9,19]. In order to provide more accurate measures of saccade size we measured saccade amplitude with an eye-tracker with a resolution of 0.5 degrees. Our results demonstrate that after rigorously controlling for saccade magnitude using high resolution eye tracking, FRPs are also modulated by task demands requiring access to working memory resources.

In line with previous findings, the results from the present study show that top-down influences of auditory cognitive load can negatively impact behavioral and neurophysiological responses in visual tasks. A study by [46] had subjects either perform a visual task in which they searched for a visual target while ignoring auditory tones or an auditory task where subjects responded to auditory oddballs tones while moving their eyes but not actively performing a visual task. While the lambda response decreased while performing the auditory task it is not clear what mechanism was responsible for the inhibition. This study differed from the current experiment in that it was not a true dual task. The inhibition observed in the lambda response by Yagi may have been due to the active inhibition of the passive task rather than a result of resource limitations. A dual task study by [47] showed decreased accuracy and increased reaction time in a target tracking task when participants were communicating over a cell phone relative to a visual-only control condition. A recent dual task ERP study by [48] had participants perform a covert visual search task while simultaneously listening to narration for later recall or while ignoring speech played backward. The results showed slower target responses and smaller visual N1 and P3 ERPs in the recall condition relative to the ignore condition. Other electrophysiological studies have shown reduced early (P1) and late (P3) visual ERPs during high, compared to low, auditory working memory load [24]. It is important to note that in many of these studies, participants were required to maintain central eye fixation throughout the experiment. Our results support and expand upon this work showing both early and late components of the FRP are affected by concurrent task demands. However, simply engaging in an auditory task does not necessarily mean visual performance will be decreased. For instance, listening to the radio does not negatively impact driving performance [47]. Our behavioral results support this finding showing that simply hearing the auditory task did not significantly affect visual task performance.

The results from the P3 FRP analysis are consistent with research [24]showing decreased amplitude as a function of auditory working memory load; however, the trend in the current study was just under statistical significance (in both 0.1 and 1Hz high-pass filtered data). We are confident, that with an increased number of target trials, the latency effect we observed in the P3 component would translate into a significant reduction in amplitude. Studies have shown a high degree of between subject variability for P3 amplitude and latency [49], which makes estimating single subject peak latency difficult using a fixed temporal window. For the analysis of P3 latency we utilized the jackknife approach along with the 50% fractional area measure, which is recommended in situations when signal to noise is low [40–42]. Here we found a statistically significant effect of auditory task load on the P3 FRP. The delayed P3 latency as a function of auditory load was largely mirrored in the behavioral data and supports prior work in the ERP literature showing a close association between P3 (specifically P3b) latency and response time [50]. The FRP epoch used in the P3 analysis exceeded our threshold of 500ms used to limit any overlapping activity from previous or subsequent saccades or fixations. It is possible that activity from a subsequent saccade and fixation overlapped with the P3 FRP of interest, reducing the signal to noise of this component. However, the average fixation duration of both targets and non-targets was greater than the observed P3 latencies, indicating signal overlap from subsequent saccades/fixations had minimal impact on the analysis.

### Interpretation of the Evoked Response in the Presence of Eye Movements

As with analyzing early components of the ERP (such as the P1) from temporally adjacent stimuli, it is also difficult to interpret EEG activity time-locked to temporally adjacent fixations due to potential overlap in the evoked response. However, as with ERPs, there are ways to either estimate the degree of overlap and remove the confounding effects or control it directly through experimental design. When the analysis window of interest consistently exceeds the fixation to saccade interval, it is possible to isolate the neural response at each fixation by applying a general linear model [19]. This is similar to the goal of the ADJAR method used in ERP analysis to correct for temporally adjacent responses [51]. Here we ensured no overlapping activity from previous or subsequent saccades by i) extending the average fixation duration via the guided search paradigm ii) constraining our analysis to include only FRPs that were not preceded or followed by saccades, fixations or blinks within 500ms of fixation onset and iii) identifying and removing independent components that covaried with saccades in the eye tracking data. Also, similar to the visually evoked P1 ERP, the lambda response is affected by low-level visual features such as luminance, contrast and spatial frequency [12–16]. Therefore, the stimuli used in the current visual search task were controlled against this low-level influence by equating their luminance (i.e., roughly the same number and values of black pixels comprising the letters within the search grid) and using low contrast 1/f noise for the background. Taken together, the results of the current study show that the lambda response and P3 components of the FRP are significantly impacted by processing resource demands and not simply low-level changes in the visual stimulus or changes in eye movement behavior.

Likewise, the lambda potential was not affected by the mere presence of auditory information as there was no difference between this response in the Silent and Ignore conditions. Furthermore, the FRP was not affected at low auditory working memory loads as the amplitude of the lambda response in 0-Back and 1-Back was not different from either the Silent or Ignore conditions. Analysis of the lambda response amplitude showed significant reduction only in the highest working memory load. This finding may be explained by considering the fixation amplifier theory, which suggests that neural sensitivity to stimulation is temporally enhanced after fixation [52]. The enhancement of neural excitability after fixation is thought to result from a phase resetting and amplification coordinated across pathways in the visual cortex [52]. It may be that the post fixation enhancement is able to overcome any effect of cognitive load up to a point. Only at high levels of working memory load does this enhancement get reduced. This would explain why the Silent, Ignore, 0-Back and 1-Back conditions showed similar lambda amplitudes but were all significantly different from the 2-Back condition. Future research should evaluate how cognitive load affects processing of visual stimuli (when actively fixated after volitional eye movements) compared with passive presentation.

### General vs Modality-Specific Cognitive Resources

Many studies have evaluated the impact of dividing attentional processing resources between unimodal and multimodal tasks. According to the multiple resource theory, processing resources can be effectively divided between multiple task modalities since each modality has an independent pool of resources to draw from (e.g. [53]. Other theories support an amodal, multipurpose pool of resources that serve multiple processing modalities [22,25,26,48]. Our results provide plausible evidence for a link between cross-modal processing resources. While the mechanism responsible for such a link cannot be pinpointed in the current study, the reduced visual processing under high auditory load suggests audition and vision share processing resources or are linked to a mechanism that does [54]. Furthermore, the results indicate that taxing processing resources in the auditory modality has a direct effect on both early and late stages of neural processing in the visual domain. If modality specific resources are indeed independent as proposed by multiple resource theory (e.g. [53] then visual task performance should not have suffered due to demands placed on the auditory system. With that said, it is difficult to draw any firm conclusion regarding the nature of the resource limited mechanism in the present study as only the auditory modality was systematically modulated. A recent study by [54] showed that when compared to an auditory only or visual only baseline, ERPs to auditory but not visual stimuli were reduced during a multisensory dual task. Future work should investigate how parametrically changing the resource demands on vision affects the FRP response as well as the auditory evoked neural response.

### Applications and Challenges for BCI

Recent studies have leveraged the P3 FRP as a primary feature to dissociate target from non-target fixations in single-trial classification providing exciting opportunities in BCI and other application spaces [6,19,29]. Many current BCIs using stimulus evoked signals for target classification rely on fixed location stimulus presentation. Using FRPs will allow for more flexible BCI applications by enabling the user to freely explore the environment, acquiring information from multiple gaze locations, thereby increasing ecological validity. Based on our results, BCIs may also be able to infer the processing demands of a given task based on FRP amplitude. Additionally, the use of FRPs in a BCI context may help evaluators determine what visual objects a user deems target relevant, without requiring an explicit a priori definition. In this way the observer would not need to directly report, either verbally or manually, when target information is fixated.

While utilizing FRPs in BCIs has great application potential, there are many challenges that need to be addressed. Many classification algorithms utilize EEG amplitude as the primary input feature and often assume a fixed temporal window when extracting amplitude information. This assumption can result in substantially reduced classification accuracy when there is high inter-trial temporal variability in the waveform. In a recent rapid serial visual presentation study (RSVP) it was shown that target P3 ERP latency was variable from trial to trial [55]. This resulted in reduced target/non-target classification accuracy as peak amplitudes in the target trials shifted away from the temporal window used in the analysis. The results of the current study show P3 FRP latencies increased with working memory load, suggesting that BCIs leveraging the FRP response may need to consider the working memory load of the operator and take corrective measures to account for latency differences in the neural signal [55].

## Conclusion

The current experiment provides new evidence that task demands imposed by auditory working memory significantly affect both early and late stages of neural processing indexed by lambda and P3 responses of the fixation-related potential. Specifically, we found smaller lambda response amplitudes and longer P3 FRP latencies under high compared to low auditory working memory load when controlling for saccadic amplitude and luminance contrast. Overall the experiment demonstrates the utility of combining synchronous recordings of EEG and eye-tracking to measure sensory and cognitive processes involved in visual search without imposing the artificial constraint of central eye fixation.

## Supporting Information

S1 Fig Saccade direction and magnitude as a function of auditory condition. A. Angular histogram of saccades in each auditory condition. B. Main sequence of saccades from all subjects for each auditory condition. C. Average saccade amplitude in each auditory condition.
(PDF)

S1 File Non-target FRP–Silent. Grand average Non-target FRP from the Silent condition.
(TXT)

S2 File Non-target FRP–Ignore. Grand average Non-target FRP from the Ignore condition.
(TXT)

S3 File Non-target FRP– 0Back. Grand average Non-target FRP from the 0Back condition.
(TXT)

S4 File Non-target FRP– 1Back. Grand average Non-target FRP from the 1Back condition.
(TXT)

S5 File Non-target FRP– 2Back. Grand average Non-target FRP from the 2Back condition.
(TXT)

S6 File Target Non-target Difference–Silent. Grand average Target Non-target difference FRP from the Silent condition.
(TXT)

S7 File Target Non-target Difference–Ignore. Grand average Target Non-target difference FRP from the Ignore condition.
(TXT)

S8 File Target Non-target Difference– 0Back. Grand average Target Non-target difference FRP from the 0Back condition.
(TXT)

S9 File Target Non-target Difference– 1Back. Grand average Target Non-target difference FRP from the 1Back condition.
(TXT)

S10 File Target Non-target Difference– 2Back. Grand average Target Non-target difference FRP from the 2Back condition.
(TXT)

S11 File Behavior and Eye Tracking Data. Behavior and eye tracking data from each subject for each condition.
(XLSX)
