# Supplementary figures and images for: The Impact of Task Demands on Fixation-Related Brain Potentials during Guided Search
Source: PLoS One. 2016 Jun 10;11(6):e0157260. doi: 10.1371/journal.pone.0157260 (PMC4902222; doi:10.1371/journal.pone.0157260)

# Supplementary A

A

Silent

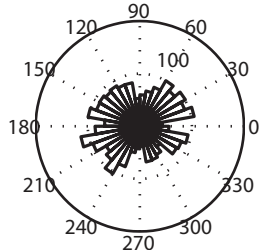

Ignore

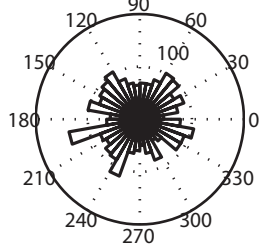

0-Back

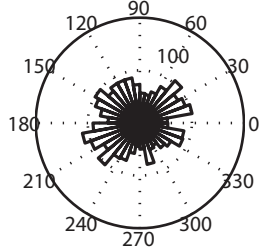

1-Back

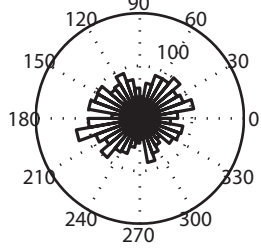

2-Back

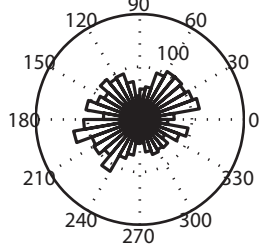

B

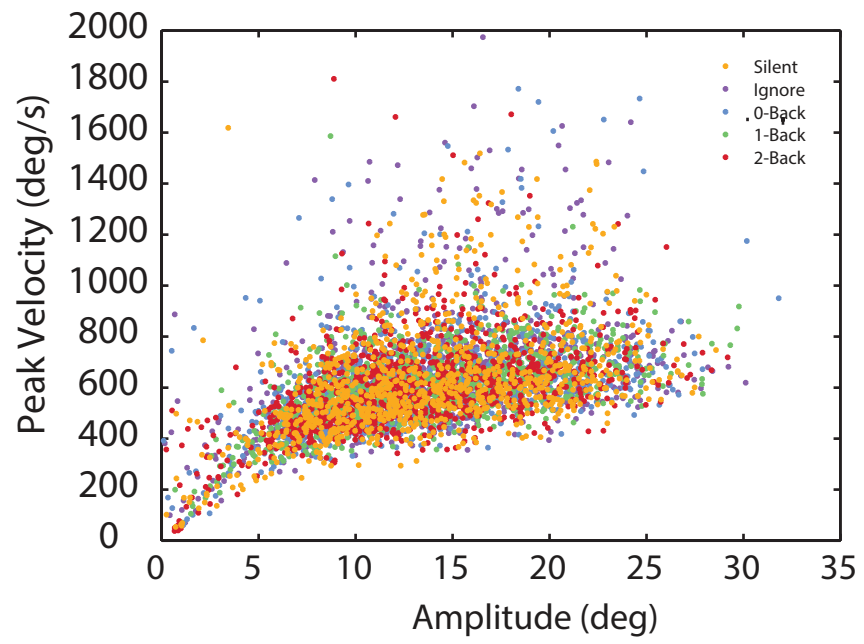

C

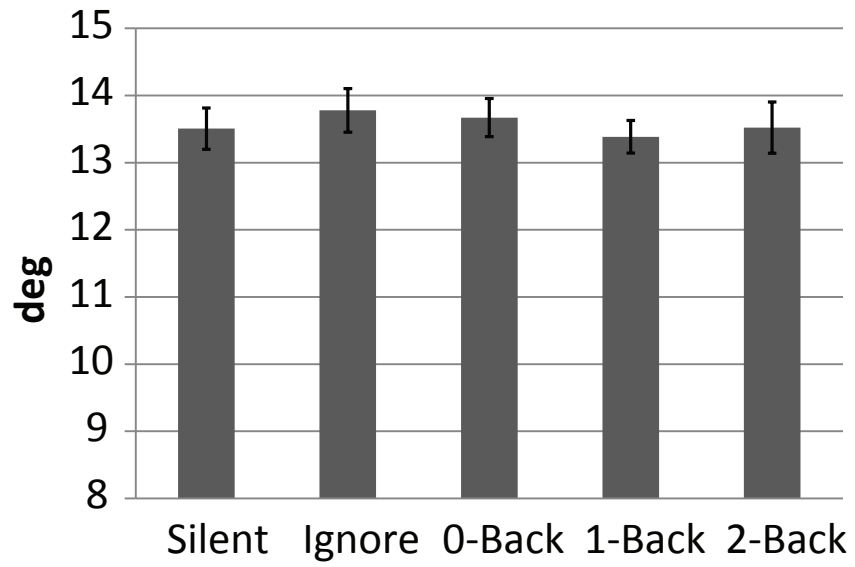

Supplement: S1 Fig — A. Angular histogram of saccades in each auditory condition. B. Main sequence of saccades from all subjects for each auditory condition. C. Average saccade amplitude in each auditory condition. (PDF) [file pone.0157260.s001.pdf]
